# Cisplatin Resistance: Genetic and Epigenetic Factors Involved

**DOI:** 10.3390/biom12101365

**Published:** 2022-09-24

**Authors:** Yuliannis Lugones, Pía Loren, Luis A. Salazar

**Affiliations:** 1 Doctoral Programme in Sciences with Major in Applied Cellular and Molecular Biology, Universidad de La Frontera, Temuco 4811230, Chile; 2 Center of Molecular Biology and Pharmacogenetics, Scientific and Technological Bioresource Nucleus, Universidad de La Frontera, Temuco 4811230, Chile

**Keywords:** cancer, cisplatin, drug resistance, epigenetics

## Abstract

Cisplatin (CDDP) is the drug of choice against different types of cancer. However, tumor cells can acquire resistance to the damage caused by cisplatin, generating genetic and epigenetic changes that lead to the generation of resistance and the activation of intrinsic resistance mechanisms in cancer cells. Among them, we can find mutations, alternative splicing, epigenetic-driven expression changes, and even post-translational modifications of proteins. However, the molecular mechanisms by which CDDP resistance develops are not clear but are believed to be multi-factorial. This article highlights a description of cisplatin, which includes action mechanism, resistance, and epigenetic factors involved in cisplatin resistance.

## 1. Introduction

Cancer is an important cause of morbidity and mortality worldwide, in every region, and irrespective of the level of human development. It has been reported that in 2020, about 9.9 million cancer deaths occurred worldwide. Studies indicate that new cancer cases will increase from 19.3 to 28.4 million by 2040 [1,2]. Cancer pathology has genetic, inflammatory, and metabolic components, which are presented by the sequential accumulation of mutations in the genome and lead to the acquisition of the tumor phenotype characterized by metabolic alterations, high proliferation rates, resistance to apoptosis, and growth factor independence, among others [3]. Cancer originates from gathering molecular alterations of genetic and/or epigenetic origin. These can be initiated by the accumulation of genetic DNA damage, affecting the DNA sequence (such as mutations and chromosomal rearrangements) or modifications in DNA, histones, and non-coding RNA that do not lead to a change in the original sequence (epigenetic modifications) [4].

Cisplatin (cis-diamminedichloroplatinum (II), CDDP) is currently the treatment of choice for many types of cancer [5,6,7,8,9,10,11]. Cisplatin exerts anticancer activity via multiple mechanisms. Its most acceptable mechanism involves the formation of DNA–platinum adducts by interacting with purine bases, activating several signal transduction pathways, and silencing or activating several genes which finally leads to apoptosis. However, side effects and drug resistance are the two inherent challenges of cisplatin that limit its application and effectiveness. The reduction of drug accumulation inside cancer cells, inactivation of drugs by reacting with glutathione and metallothioneins, and faster repairing of DNA lesions are responsible for cisplatin resistance [12].

In addition, several studies have demonstrated the relationship between chemotherapeutic resistance and the epigenetic processes associated with DNA and histone modifications, and gene expression regulation. This review summarizes the mechanism of action and resistance to cisplatin, and the epigenetic factors associated with it, given the importance of finding new biomarkers for chemotherapeutic resistance.

## 2. Cisplatin: Mechanism of Action

Cisplatin is a neutral coordination complex with a central platinum (II) atom bonded to two chloride atoms and two ammonia molecules in the cis position. The coordinated covalent bonds of platinum with nitrogen are virtually irreversible, but their bonds with chloride ligands, in aqueous media and under certain pH and temperature conditions, are highly labile [13].

Cisplatin’s mechanism of action is initiated by the activation of the complex in the intracellular medium by the hydrolysis of chloride molecules. The cisplatin molecule hydrolyzes in the cytoplasm, and acts as a potent electrophilic agent, reacting with nucleic acids and sulfhydryl groups of proteins [14,15]. However, the therapeutic target of this drug is genomic and mitochondrial DNA. The covalent binding of CDDP to DNA via platinum atoms, by intercalating between base pairs (mainly purines), generates so-called cisplatin–DNA adducts. Platinum binds mainly through nitrogen at position 7 of the imidazole ring of the guanine and adenine of the corresponding DNA nucleotides (2′-deoxyadenosine 5′-monophosphate, dAMP; and 2′-deoxyguanosine 5′-monophosphate, dGMP) since these are the atoms with the highest electron density, and are most accessible to electrophilic attack by cisplatin. Moreover, binding is particularly favored with guanines located in the major groove of the DNA double helix [14,16,17]. As a consequence of the formation of these DNA adducts, the DNA replication mechanisms will be inhibited and therefore effect its transcription processes [13]. In response to this cellular damage, signaling pathways will be activated that will lead in the first instance to cell cycle arrest through the action of the tumor suppressor protein p53 in an attempt to repair the damaged DNA [18,19]. Subsequently, cell death by apoptosis occurs mediated by proteins such as Bcl-2 if the DNA damage is not repaired [14,18].

## 3. Resistance to Cisplatin Treatment

### 3.1. Mechanisms of Cisplatin Resistance

The development of chemotherapeutic resistance is a problem of great importance despite great advances in understanding the molecular mechanisms of cancer [20,21]. It has been observed that 50% of patients treated with cisplatin either go on to develop intrinsic resistance or acquire multidrug resistance rapidly [13,22,23]. In both cases, the mechanisms of resistance are based on a reduction in the accumulation of cytotoxic compounds in the cytosol of cancer cells, together with the activation of DNA repair mechanisms that protect cancer cells from potentially lethal stresses caused by chemo drugs [24].

A cell population is considered to be resistant when it increases its baseline tolerance, managing to proliferate in a medium with twice, or more than twice, the drug concentration tolerated by the parental line, for which mechanisms are activated that allow it to avoid drug-induced cell death, which is related to morphological variations described as an increase in cell size, increase in the nucleus–cytoplasmic ratio, irregularities in the cell membrane borders, or an increase in cytoplasmic granules [25,26,27].

Resistance to CDDP and other chemo drugs are directly related to the stage of tumor progression because cancer cells acquire additional genetic and epigenetic alterations that confer growth advantages, such as proliferation, and consequently, the expected cytotoxic or cytostatic effect does not occur [28]. Both mutations and changes in gene expression and post-translational modifications of proteins are some of the alterations that have been associated with the acquisition of resistance to these drugs [27,29,30].

Several factors are involved in cisplatin resistance and can be classified as pre-target resistance, on-target resistance, post-target resistance, and off-target resistance [16,31].

#### 3.1.1. Pre-Target Resistance

Pre-target resistance is related to the reduction of CDDP entry into the cell or to a more significant expulsion of CDDP into the extracellular space [16,31]. CDDP is a very polar molecule and enters cells relatively slowly compared to other molecules used for cancer treatment. CDDP entry into the cell is influenced by the concentrations of sodium and potassium ions, pH, the presence of reducing agents, and the action of transporters and channels, which are coupled to the passive diffusion mechanism [16]. Among the proposed transporters, the organic cation transporters (OCT) and the copper transporter protein CTR1 (copper transport protein 1) stand out. It was observed that cisplatin causes a decrease in the expression of these transporter proteins, decreasing the concentration of the drug inside the cells as a mechanism of resistance [32,33,34,35,36]. On the other hand, some studies suggest that the transporter proteins ATP7A and ATP7B and the multidrug resistance-associated protein MRP2 may also be involved in CDDP resistance by increasing the flux of CDDP out of the cell [35,37,38,39]. Another “pre-target” mechanism refers to the intracellular inactivation of cisplatin by the formation of complexes with compounds present in the cell cytosol, mainly those containing thiol groups such as reduced glutathione (GSH) or metallothioneins. This process occurs in the cytoplasm where cisplatin is a potent electrophilic agent that acts with these nucleophilic groups and thus decreases drug interactions with DNA [40,41].

#### 3.1.2. On-Target Resistance

On-target resistance involves processes related to molecular damage caused by cisplatin to DNA [16,31]. Once CDDP is bound to DNA, the cell can survive by activating DNA repair mechanisms or by tolerance to genetic damage. Nucleotide excision repair is the first pathway that begins to repair DNA in the face of cisplatin resistance. This repair pathway is responsible for removing the bonds formed between platinum and DNA. Once CDDP binds to DNA, the cell can survive by activating DNA repair mechanisms or by tolerance to genetic damage. Within the DNA repair pathways, nucleotide excision repair appears to play a key role in eliminating cisplatin damage. This repair pathway is responsible for eliminating the bonds formed between platinum and DNA through the action of ERCC1 (excision repair cross-complementing 1) and XPF (Xeroderma pigmentosum complementation group F) proteins. These proteins form a heterodimer and act by cutting the 5′ end of the area of the strand where the platinum has bound to the DNA to allow subsequent elimination of the adduct. A relationship between increased expression levels of ERCC1 endonuclease and CDDP resistance has been described in different cell lines and patient samples [42,43,44,45,46]. In addition, increased tolerance to cisplatin-induced damage may be related to a loss of function of the mispaired base repair (MMR) pathway. During MMR, different proteins recognize intracatenary adducts, including MSH2 and MLH1, which, together with other MMR proteins, detect damage and transmit proapoptotic signals. MSH2 and MLH1 genes have been mutated or downregulated due to CDDP resistance, resulting in the inhibition of apoptosis [16]. On the other hand, cisplatin induces intercatenary adducts that are usually repaired by the homologous recombination mechanism (HRR). In breast and ovarian cancer, the BRCA1 and BRCA2 genes, which code for proteins of the HRR system, have been found to be mutated [47]. In particular, cancers deficient in the HRR system have a different phenotype and are often more sensitive to cisplatin than their counterparts in which the HRR mechanism functions usually [48]. Finally, it should be mentioned that damage tolerance is related to the replicative by-pass of CDDP-induced injury that certain classes of polymerases, such as β, ƞ, and ζ, can perform. This results in DNA synthesis not being blocked and, consequently, apoptotic pathways are not activated [16,49,50].

#### 3.1.3. Post-Target Resistance

**Figure 1:**
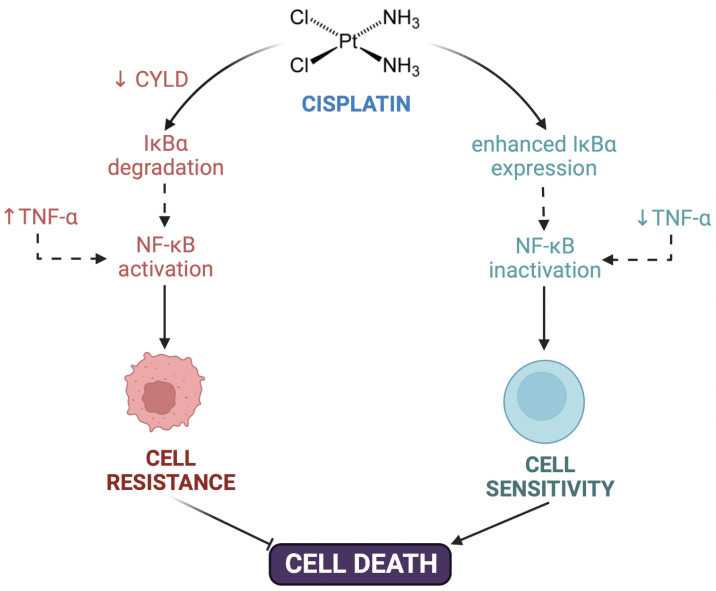
Contribution of NF-κΒ to cisplatin resistance. Cisplatin resistance is caused by downregulation of CYLD lysine 63 deubiquitinase (CYLD), triggering the suppression of cell death via NF-κB hyperactivation. TNF-α also contributed to NF-κΒ activation and cell resistance. Created with Biorender.com.

Post-target resistance includes mechanisms that affect signaling pathways leading to cell death triggered by adducts [16,31]. Among these mechanisms is the inactivation of the TP53 gene, which produces a loss of apoptotic activity and the appearance of resistance in 50% of human cancers [51]. TP53 encodes for the p53 protein, which induces apoptosis by activating the signaling cascade to effector molecules such as Bax (BCL2-associated X protein). Similarly, the inactivation of caspases such as caspases 3, 8, and 9, of great importance in apoptosis, has been associated with resistance to cisplatin in different types of cancers such as head and neck, ovarian, breast, and others [52,53,54,55,56]. Cisplatin resistance is also caused by CYLD (CYLD lysine 63 deubiquitinase) downregulation, which triggers the reduction of intracellular CDDP accumulation and the suppression of cell death via NF-κB hyperactivation [57]. TNF-α also contributes to NF-κΒ activation in head and neck cancer cells [58]. Even more, the inhibition of both NF-κΒ and MAPK/HO-1 signaling pathways also reduce oxidative stress and CDDP-induced resistance in non-small cell lung cancer [59] (Figure 1).

#### 3.1.4. Off-Target Resistance

Off-target resistance is related to alterations in signaling pathways that are not directly related to cisplatin but interfere with cisplatin-induced proapoptotic events [16,31]. This type of mechanism includes the overexpression of the proto-oncogene ERBB2 that encodes for the HER2 (human epidermal growth factor receptor) protein, and the gene encoding the DYRK1B (dual specificity tyrosine phosphorylation regulated kinase 1B) kinase. The former is key to activating numerous signaling pathways that regulate functions such as cell differentiation, growth, and survival [60]. The second facilitates cell survival by increasing the activity of antioxidant enzymes such as ferroxidase and superoxide dismutase, which constitute the defense of cells against oxidative stress [61]. There are also several mechanisms associated with the organism’s response to stressful situations or poorly characterized ones related to resistance to cisplatin, including autophagy (a cellular process responsible for the degradation and recycling of damaged cellular components) [62,63]. In this sense, different studies postulate that the inhibition of autophagy can restore cell sensitivity to cisplatin, at least in vitro [63].

**Figure 2:**
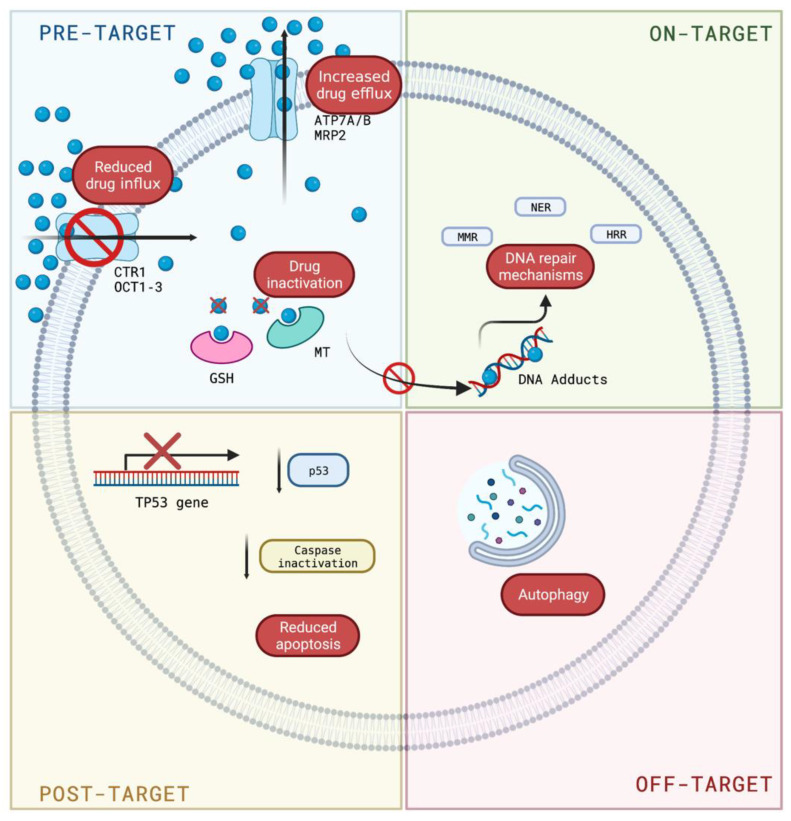
Mechanisms of resistance to cisplatin. Pre-target resistance related to the control of the entry or exit of cisplatin into the cell; on-target resistance implicates mechanisms involved in DNA damage; post-target resistance includes mechanisms that interfere with cell death caused by DNA adducts; and off-target resistance is related to cisplatin-induced proapoptotic events. Blue circle: cisplatin. Created with Biorender.com (https://biorender.com/, accessed on 1 August 2022).

Figure 2 summarizes the mechanisms of resistance to cisplatin and, although they have been grouped into several groups for better study and understanding, they should not be considered as isolated events but depend on the simultaneous activation of several molecular mechanisms that ultimately lead to chemoresistance.

## 4. Epigenetics and Resistance to Treatment

Cisplatin resistance is multifactorial and cannot be explained by the deregulation of a single molecular mechanism. This is a major obstacle to avoiding cisplatin resistance, and one of the main problems associated with its use. However, current studies suggest that resistance to cisplatin treatment may also be mediated by epigenetic factors that modify the expression of genes important in the response to the drug (Table 1).

Epigenetics refers to heritable changes in gene expression that are not attributable to variations in DNA sequence. In other words, epigenetics is based on the study of DNA methylation, histone modifications, and the regulation of gene expression by non-coding RNAs as epigenetic mechanisms [64,65,66,67].

### 4.1. Epigenetic Mechanisms Associated with Pre-Target, on-Target, and Post-Target Resistance

The importance of studying the relationship that seems to exist between the epigenetic modifications of the promoters of non-coding RNAs and the development of phenotypes resistant to chemotherapeutic drugs, such as cisplatin, has grown enormously in recent years. Among regulatory mechanisms of miRNAs expression, we found silencing due to the methylation of their regulatory regions resulting in the overexpression of their target genes [68,69]. An example of how miRNAs are related to the pre-target resistance mechanism is found with miR-38. The inhibition of miR-38 desensitizes breast cancer cells to cisplatin through the expression of ABCB1/MDR1 mRNA. ABCB1/MDR1 are cytoplasmic membrane transporter proteins related to resistance to chemotherapeutics by preventing the intracellular concentration of this drug [70]. Similarly, miR-148a inhibits the expression of ATP7A, another transporter protein involved in cisplatin resistance, which may accelerate chemotherapy-induced apoptosis in breast cancer cells [12]. A lncRNA, ROR, has also been found to be related to cisplatin transport in osteosarcoma, regulating miR-153-3p/ABCB1 expression [71], whereas deletion of miR-200c causes resistance to platinum-derived drugs by targeting the DNA repair proteins ERCC3 and ERCC4 in gastric cancer as a resistance mechanism related to DNA damage (on-target resistance) [72].

As mentioned above, post-target resistance mechanisms include processes that interfere with cell death caused by the formation of cisplatin adducts. Hypermethylation of the miR-200b promoter is an example of this fact. In bladder cancer, miR-200b methylation increases the expression of genes associated with chemosensitivity and apoptosis such as IGFBP3, ICAM1, and TNFSF10 genes, leading to cisplatin resistance. Therefore, miR-200b could be a biomarker associated with chemoresistance and a therapeutic target for patients who develop resistance in this type of cancer [73]. Shindo et al. demonstrated that in ovarian tumors, miR-100 and miR-214 have been negatively regulated, the latter targeting the tumor suppressor gene PTEN associated with platinum resistance. miR-214 inhibits PTEN transduction and activates the Akt pathway, inducing cell survival and cisplatin resistance [74]. A similar mechanism is shared by ROR where drug resistance occurs via apoptosis, but in this case, ROR is a negative regulator of p53 and the PI3K/Akt/mTOR signaling pathway in nasopharyngeal and lung carcinoma, respectively [75,76]. Likewise, SNHG15, p53-regulated lncRNA can suppress cisplatin-induced apoptosis through miR-335-3p [77]. Another potential therapeutic target in the treatment of cisplatin-resistant ovarian cancer is miR-335-5p, which enhances the sensitivity to the chemotherapeutic by increased expression of BCL2L2 when miRNA is overexpressed [78].

The DNA methylation process in tumor cells inhibits specific genes necessary under normal conditions for proper cell function [79]. Platinum-based chemotherapy was found to contribute to the modification of DNA methylation in cancer [80]. The loss of IGFBP-3 (insulin-like growth factor binding protein-3) gene expression in NSCLC (non-small cell lung cancer) can activate the IGF-IR/PI3K/AKT survival pathway, as an effect produced by CDDP administration. The silencing of this gene is produced by the hypermethylation of its promoter in cisplatin-resistant cell phenotypes, indicating that the methylation of the IGFBP-3 promoter is mediating the emergence of resistance to this drug [81].

### 4.2. Epigenetic Mechanisms Associated with Off-Target Resistance

In off-target cisplatin resistance, we also found epigenetic mechanisms that are related to drug-induced proapoptotic events. Multiple studies confirm that cancer cells take advantage of stem cell properties to form cancer stem cells through DNA methylation processes [82,83,84]. In NSCLC, cancer stem cells are associated with resistance to chemotherapy and in particular to cisplatin [84,85]. An example of this is Gli1, whose drug resistance is due to the upregulation of Sox2, favoring self-renewal in NSCLC cancer stem cells [86]. Studies of forkhead box F1 (FOXF1) link its decreased expression with advancing tumorigenesis [87,88]. In A549/DDP cells treated with cisplatin, FOXF1 transcription is favored by demethylation of the regulatory region of the FOXF1 gene. In turn, FOXF1 promotes drug resistance by promoting cancer stem cell properties in NSCLC [89]. In metabolic enzymes, the effect of DNA methylation has also been studied, demonstrating its alteration in cisplatin-resistant cells. The enzymes spermidine/spermine N1-acetyltransferase (SAT1) and argininosuccinate synthase 1 (ASS1), in T24 bladder cancer cells, are decreased due to epigenetic silencing of the genes encoding it (genes for polyamine and amino acid metabolism catalysts, respectively) [90]. As another example, there is the enzyme NAGA (α-N-acetylgalactosaminidase), responsible for the activation of the Gc macrophage activating factor (GcMAF), whose promoter hypermethylation produces resistance to cisplatin [91].

Alterations in the expression of histone deacetylases and demethylases also contribute to developing resistance to cisplatin in certain types of cancer. An example of this occurs in NSCLC, in which the increased expression of these enzymes, specifically histone-deacetylase-6 (HDAC6), generates resistant phenotypes and decreases apoptosis in these cells [92]. On the other hand, oxidative stress caused by cisplatin also induces changes at the level of histone demethylases, which alter histone methylation patterns and constitute a gene silencing mechanism in some types of cancer [93].

Dysregulation of miR-7, miR-132, and miR-148a has also been associated with cisplatin resistance in ovarian tumor cells and also in lung cancer. MiR-7 directly regulates the action of MAFG (the musculoaponeurotic fibrosarcoma oncogene family, protein G), which is overexpressed in platinum resistance in cancer cell lines. MAFG is associated with detoxification in the face of oxidative stress, protecting against free radicals generated by the cell when cisplatin is administered [94]. Meanwhile, miR-132 and miR-148a target TGF-β1 and WNT10b, respectively, regulating migration and invasion in cisplatin-resistant oral squamous cell carcinoma and colorectal cancer [95,96].

Of lncRNAs and their resistance to cisplatin, it has recently been known that differential expression in response to therapy is more frequent in cis-acting lncRNAs compared to overlapping ones, whereas significantly altered methylation profiles were more commonly associated with overlapping lncRNAs. Another rationale is that overlapping lncRNAs present a higher amount of CpG islands (CGIs) shared with most of their associated coding genes [97]. Similarly, studies have described the relationship between the expression of lncRNAs and the occurrence of resistance in different tumors. Studies show that the lncRNA HOTTIP, a regulator of the transcription of genes of the HOXA family, is associated with resistance to chemotherapy in osteosarcoma [98,99]. UCA1 is another lncRNA associated with resistance to platinum-derived compounds in bladder and tongue cancer. UCA1 may enhance cisplatin resistance in tongue cancer cells by regulating autophagy signaling [100,101]. Similarly, a study of the long non-coding RNA taurine-regulated gene 1 (TUG1) demonstrated that upregulated TUG1 confers cisplatin resistance in esophageal squamous cell carcinoma by epigenetically suppressing PDCD4 expression through EZH2 [102].

In summary, the main genetic and epigenetic factors related to cisplatin resistance can be summarized in Table 1.

**Table 1:** Factors regulating genetic and epigenetic mechanisms during cisplatin resistance.

Type	Cisplatin Resistance	Molecule Involved	Reference
Genetic factors	Pre-target	Decreased CTR1 expression	[32,33,34,35,36]
		Enhanced ATP7A, ATP7B, and MRP2 expression	[35,37,38,39]
		Intracellular inactivation of cisplatin by GSH or metallothioneins	[40,41]
	On-target	Enhanced ERCC1 endonuclease	[42,43,44,45,46]
		Downregulation of MSH2 and MLH1 expression	[16]
		Enhanced homologous recombination mechanism	[48]
		Augmented polymerases β, η, & ζ activity	[16,49,50]
	Post-target	Inactivation of TP53 gene	[51]
		Inactivation of caspases	[52,53,54,55,56]
		CYLD Lysine 63 deubiquitinase downregulation	[57]
		NF-κB hyperactivation	[57]
	Off-target	Overexpression of human epidermal growth factor receptor and dual specificity tyrosine phosphorylation regulated kinase 1B	[60]
		Enhanced antioxidant enzymes such as ferroxidase and superoxide dismutase	[61]
		Autophagy	[62,63]
Epigenetic factors	Pre-target	miR-38 (regulating ABCB1/MDR1)	[70]
		miR-148a (regulating ATP7A)	[12]
		lncRNA ROR (targeting miR-153-3p/ABCB1)	[71]
	On target	miR-200c (targeting ERCC3/ERCC4)	[72]
	Post-target	Methylation of miR-200b enhances IGFBP3, ICAM1, and TNFSF10 gene expression	[73]
		Downregulation of miR-100 and miR-214 (targeting PTEN)	[74]
		LncRNA ROR (targeting TP53)	[75,76]
		miR-335-3p (regulates apoptosis)	[77]
	Off-target	Demethylation of the regulatory region of the FOXF1 gene	[89]
		Epigenetic silencing of spermidine/spermine N1-acetyltransferase and argininosuccinate synthase 1	[90]
		Enhanced HDAC6 expression	[92]
		Dysregulation of miR-7, miR-132, and miR-148a	[94,95,96]
		Overexpression of lncRNA HOTTIP activates Wnt/β-catenin pathway	[98,99]
		LncRNA UCA1 promotes autophagy	[100,101]
		LncRNA TUG1 suppress PDCD4 expression	[102]

## 5. Conclusions

Resistance to platinum-based drugs is not only a problem in the face of treatment with chemotherapeutics, but also a break in the fight against cancer. However, the genes whose promoters are hypermethylated in cancer and which are related to cisplatin resistance as a consequence of the epigenetic silencing to which they are subjected are becoming increasingly well known. Knowledge of epigenetic regulation in cancer drug resistance will contribute to developing biomarkers and cancer therapies.

## Data Availability

Not applicable.

## References

[1] Sung H., Ferlay J., Siegel R.L., Laversanne M., Soerjomataram I., Jemal A., Bray F. (2021). Global cancer statistics 2020: GLOBOCAN estimates of incidence and mortality worldwide for 36 cancers in 185 countries. CA Cancer J. Clin..

[2] WHO (2021). World Health Statistics 2021: Monitoring Health for the SDGs, Sustainable Development Goals.

[3] Hanahan D., Weinberg R.A. (2011). Hallmarks of cancer: The next generation. Cell.

[4] Flavahan W.A., Gaskell E., Bernstein B.E. (2017). Epigenetic plasticity and the hallmarks of cancer. Science.

[5] Vermorken J.B., Remenar E., Van Herpen C., Gorlia T., Mesia R., Degardin M., Stewart J.S., Jelic S., Betka J., Preiss J.H. (2007). Cisplatin, fluorouracil, and docetaxel in unresectable head and neck cancer. N. Engl. J. Med..

[6] Motzer R.J., Sheinfeld J., Mazumdar M., Bajorin D.F., Bosl G.J., Herr H., Lyn P., Vlamis V. (1995). Etoposide and cisplatin adjuvant therapy for patients with pathologic stage II germ cell tumors. J. Clin. Oncol..

[7] Li D., Zhang Y., Xie Y., Xiang J., Zhu Y., Yang J. (2013). Enhanced tumor suppression by adenoviral PTEN gene therapy combined with cisplatin chemotherapy in small-cell lung cancer. Cancer Gene Ther..

[8] Magali L., Pascal F., Serge A., Mathieu B., Ayoube Z., Claire T., Christiane M. (2020). Better survival in impaired renal function patients with metastatic non-small cell lung cancer treated by cisplatin-pemetrexed. Eur. J. Clin. Pharmacol..

[9] Armstrong D.K., Bundy B., Wenzel L., Huang H.Q., Baergen R., Lele S., Copeland L.J., Walker J.L., Burger R.A. (2006). Intraperitoneal cisplatin and paclitaxel in ovarian cancer. N. Engl. J. Med..

[10] Moore K.N., Herzog T.J., Lewin S., Giuntoli R.L., Armstrong D.K., Rocconi R.P., Spannuth W.A., Gold M.A. (2007). A comparison of cisplatin/paclitaxel and carboplatin/paclitaxel in stage IVB, recurrent or persistent cervical cancer. Gynecol. Oncol..

[11] Coppin C., Gospodarowicz M.K., James K., Tannock I.F., Zee B., Carson J., Pater J., Sullivan L.D. (1996). Improved local control of invasive bladder cancer by concurrent cisplatin and preoperative or definitive radiation. The National Cancer Institute of Canada Clinical Trials Group. J. Clin. Oncol..

[12] Yu Z., Cao W., Ren Y., Zhang Q., Liu J. (2020). ATPase copper transporter A, negatively regulated by miR-148a-3p, contributes to cisplatin resistance in breast cancer cells. Clin. Transl. Med..

[13] Dasari S., Tchounwou P.B. (2014). Cisplatin in cancer therapy: Molecular mechanisms of action. Eur. J. Pharmacol..

[14] Basu A., Krishnamurthy S. (2010). Cellular responses to Cisplatin-induced DNA damage. J. Nucleic Acids.

[15] Florea A.-M., Büsselberg D. (2011). Cisplatin as an anti-tumor drug: Cellular mechanisms of activity, drug resistance and induced side effects. Cancers.

[16] Galluzzi L., Senovilla L., Vitale I., Michels J., Martins I., Kepp O., Castedo M., Kroemer G. (2012). Molecular mechanisms of cisplatin resistance. Oncogene.

[17] Shen D.-W., Pouliot L.M., Hall M.D., Gottesman M.M. (2012). Cisplatin resistance: A cellular self-defense mechanism resulting from multiple epigenetic and genetic changes. Pharmacol. Rev..

[18] Siddik Z.H. (2003). Cisplatin: Mode of cytotoxic action and molecular basis of resistance. Oncogene.

[19] Fuertes M., Castilla J., Alonso C., Prez J. (2003). Cisplatin biochemical mechanism of action: From cytotoxicity to induction of cell death through interconnections between apoptotic and necrotic pathways. Curr. Med. Chem..

[20] Kartal-Yandim M., Adan-Gokbulut A., Baran Y. (2016). Molecular mechanisms of drug resistance and its reversal in cancer. Crit. Rev. Biotechnol..

[21] Steding C.E. (2016). Creating chemotherapeutic-resistant breast cancer cell lines: Advances and future perspectives. Future Oncol..

[22] Pogribny I.P., Filkowski J.N., Tryndyak V.P., Golubov A., Shpyleva S.I., Kovalchuk O. (2010). Alterations of microRNAs and their targets are associated with acquired resistance of MCF-7 breast cancer cells to cisplatin. Int. J. Cancer.

[23] Velasco G., Sánchez C., Guzmán M. (2016). Anticancer mechanisms of cannabinoids. Curr. Oncol..

[24] Aye Y., Li M., Long M., Weiss R. (2015). Ribonucleotide reductase and cancer: Biological mechanisms and targeted therapies. Oncogene.

[25] McDermott M., Eustace A., Busschots S., Breen L., Clynes M., O’Donovan N., Stordal B. (2014). In vitro development of chemotherapy and targeted therapy drug-resistant cancer cell lines: A practical guide with case studies. Front. Oncol..

[26] Lukyanova N.Y., Rusetskya N., Tregubova N., Chekhun V. (2009). Molecular profile and cell cycle in MCF-7 cells resistant to cisplatin and doxorubicin. Exp. Oncol..

[27] Puspita N.A., Bedford A. (2017). Morphological Changes of Cisplatin-resistant Human Breast Cancer MCF-7 Cell Line. Int. J. Integr. Health Sci..

[28] Hinojosa-García L.M., Dueñas-González A. (2000). Papel de la quimioterapia en el tratamiento del carcinoma cervicouterino. Rev. Inst. Nal. Cancerol. (Mex).

[29] Aleksakhina S.N., Kashyap A., Imyanitov E.N. (2019). Mechanisms of acquired tumor drug resistance. Biochim. Biophys. Acta (BBA)-Rev. Cancer.

[30] Lund R.J., Huhtinen K., Salmi J., Rantala J., Nguyen E.V., Moulder R., Goodlett D.R., Lahesmaa R., Carpén O. (2017). DNA methylation and transcriptome changes associated with cisplatin resistance in ovarian cancer. Sci. Rep..

[31] Galluzzi L., Vitale I., Michels J., Brenner C., Szabadkai G., Harel-Bellan A., Castedo M., Kroemer G. (2014). Systems biology of cisplatin resistance: Past, present and future. Cell Death Dis..

[32] Müller J., Lips K.S., Metzner L., Neubert R.H., Koepsell H., Brandsch M. (2005). Drug specificity and intestinal membrane localization of human organic cation transporters (OCT). Biochem. Pharmacol..

[33] Ciarimboli G., Bonetti A., Leone R., Muggia F.M., Howell S.B. (2009). Organic Cation Transporters 2 as Mediators of Cisplatin Nephrotoxicity. Platinum and Other Heavy Metal Compounds in Cancer Chemotherapy.

[34] Safaei R. (2006). Role of copper transporters in the uptake and efflux of platinum containing drugs. Cancer Lett..

[35] Kuo M.T., Fu S., Savaraj N., Chen H.H. (2012). Role of the human high-affinity copper transporter in copper homeostasis regulation and cisplatin sensitivity in cancer chemotherapy. Cancer Res..

[36] Köberle B., Brenner W., Albers A., Usanova S., Thüroff J.W., Kaina B. (2010). ERCC1 and XPF expression in human testicular germ cell tumors. Oncol. Rep..

[37] Liang Z.D., Long Y., Tsai W.-B., Fu S., Kurzrock R., Gagea-Iurascu M., Zhang F., Chen H.H., Hennessy B.T., Mills G.B. (2012). Mechanistic basis for overcoming platinum resistance using copper chelating agents. Mol. Cancer Ther..

[38] Korita P.V., Wakai T., Shirai Y., Matsuda Y., Sakata J., Takamura M., Yano M., Sanpei A., Aoyagi Y., Hatakeyama K. (2010). Multidrug resistance-associated protein 2 determines the efficacy of cisplatin in patients with hepatocellular carcinoma. Oncol. Rep..

[39] Yamasaki M., Makino T., Masuzawa T., Kurokawa Y., Miyata H., Takiguchi S., Nakajima K., Fujiwara Y., Matsuura N., Mori M. (2011). Role of multidrug resistance protein 2 (MRP2) in chemoresistance and clinical outcome in oesophageal squamous cell carcinoma. Br. J. Cancer.

[40] Chen H.H., Kuo M.T. (2010). Role of glutathione in the regulation of Cisplatin resistance in cancer chemotherapy. Met.-Based Drugs.

[41] Knipp M., Karotki A.V., Chesnov S., Natile G., Sadler P.J., Brabec V., Vašák M. (2007). Reaction of Zn7Metallothionein with cis-and trans-[Pt (N-donor) 2Cl2] anticancer complexes: Trans-PtII complexes retain their N-donor ligands. J. Med. Chem..

[42] Martin L.P., Hamilton T.C., Schilder R.J. (2008). Platinum resistance: The role of DNA repair pathways. Clin. Cancer Res..

[43] Saldivar J.S., Wu X., Follen M., Gershenson D. (2007). Nucleotide excision repair pathway review I: Implications in ovarian cancer and platinum sensitivity. Gynecol. Oncol..

[44] Chang I.-Y., Kim M.-H., Kim H.B., Kim S.-H., Kim H.-Y., You H.J. (2005). Small interfering RNA-induced suppression of ERCC1 enhances sensitivity of human cancer cells to cisplatin. Biochem. Biophys. Res. Commun..

[45] Usanova S., Piée-Staffa A., Sied U., Thomale J., Schneider A., Kaina B., Köberle B. (2010). Cisplatin sensitivity of testis tumour cells is due to deficiency in interstrand-crosslink repair and low ERCC1-XPF expression. Mol. Cancer.

[46] Hirakawa M., Sato Y., Ohnuma H., Takayama T., Sagawa T., Nobuoka T., Harada K., Miyamoto H., Sato Y., Takahashi Y. (2013). A phase II study of neoadjuvant combination chemotherapy with docetaxel, cisplatin, and S-1 for locally advanced resectable gastric cancer: Nucleotide excision repair (NER) as potential chemoresistance marker. Cancer Chemother. Pharmacol..

[47] Narod S.A., Foulkes W.D. (2004). BRCA1 and BRCA2: 1994 and beyond. Nat. Rev. Cancer.

[48] Farmer H., McCabe N., Lord C.J., Tutt A.N., Johnson D.A., Richardson T.B., Santarosa M., Dillon K.J., Hickson I., Knights C. (2005). Targeting the DNA repair defect in BRCA mutant cells as a therapeutic strategy. Nature.

[49] Bassett E., Vaisman A., Tropea K.A., McCall C.M., Masutani C., Hanaoka F., Chaney S.G. (2002). Frameshifts and deletions during in vitro translesion synthesis past Pt–DNA adducts by DNA polymerases β and η. DNA Repair.

[50] Albertella M.R., Green C.M., Lehmann A.R., O’Connor M.J. (2005). A role for polymerase η in the cellular tolerance to cisplatin-induced damage. Cancer Res..

[51] Martinez-Rivera M., Siddik Z.H. (2012). Resistance and gain-of-resistance phenotypes in cancers harboring wild-type p53. Biochem. Pharmacol..

[52] Ding Z., Yang X., Pater A., Tang S.-C. (2000). Resistance to apoptosis is correlated with the reduced caspase-3 activation and enhanced expression of antiapoptotic proteins in human cervical multidrug-resistant cells. Biochem. Biophys. Res. Commun..

[53] Duiker E.W., Meijer A., van der Bilt A.R., Meersma G.J., Kooi N., van der Zee A.G., De Vries E., de Jong S. (2011). Drug-induced caspase 8 upregulation sensitises cisplatin-resistant ovarian carcinoma cells to rhTRAIL-induced apoptosis. Br. J. Cancer.

[54] Kim P.K., Mahidhara R., Seol D.-W. (2001). The role of caspase-8 in resistance to cancer chemotherapy. Drug Resist. Updates.

[55] Kuwahara D., Tsutsumi K., Oyake D., Ohta T., Nishikawa H., Koizuka I. (2003). Inhibition of caspase-9 activity and Apaf-1 expression in cisplatin-resistant head and neck squamous cell carcinoma cells. Auris Nasus Larynx.

[56] Nikounezhad N., Nakhjavani M., Shirazi F.H. (2016). Generation of cisplatin-resistant ovarian cancer cell lines. Iran. J. Pharm. Sci..

[57] Suenaga N., Kuramitsu M., Komure K., Kanemaru A., Takano K., Ozeki K., Nishimura Y., Yoshida R., Nakayama H., Shinriki S. (2019). Loss of Tumor Suppressor CYLD Expression Triggers Cisplatin Resistance in Oral Squamous Cell Carcinoma. Int. J. Mol. Sci..

[58] Kim S.B., Kim J.S., Lee J.H., Yoon W.J., Lee D.S., Ko M.S., Kwon B.S., Choi D.H., Cho H.R., Lee B.J. (2006). NF-kappaB activation is required for cisplatin-induced apoptosis in head and neck squamous carcinoma cells. FEBS Lett..

[59] Wang L.H., Li Y., Yang S.N., Wang F.Y., Hou Y., Cui W., Chen K., Cao Q., Wang S., Zhang T.Y. (2014). Gambogic acid synergistically potentiates cisplatin-induced apoptosis in non-small-cell lung cancer through suppressing NF-kappaB and MAPK/HO-1 signalling. Br. J. Cancer.

[60] Deng X., Ewton D.Z., Friedman E. (2009). Mirk/Dyrk1B maintains the viability of quiescent pancreatic cancer cells by reducing levels of reactive oxygen species. Cancer Res..

[61] Fijołek J., Wiatr E., Rowińska-Zakrzewska E., Giedronowicz D., Langfort R., Chabowski M., Orłowski T., Roszkowski K. (2006). p53 and HER2/neu expression in relation to chemotherapy response in patients with non-small cell lung cancer. Int. J. Biol. Markers.

[62] Sui X., Chen R., Wang Z., Huang Z., Kong N., Zhang M., Han W., Lou F., Yang J., Zhang Q. (2013). Autophagy and chemotherapy resistance: A promising therapeutic target for cancer treatment. Cell Death Dis..

[63] Ren J.-H., He W.-S., Nong L., Zhu Q.-Y., Hu K., Zhang R.-G., Huang L.-L., Zhu F., Wu G. (2010). Acquired cisplatin resistance in human lung adenocarcinoma cells is associated with enhanced autophagy. Cancer Biother. Radiopharm..

[64] Wei J.-W., Huang K., Yang C., Kang C.-S. (2017). Non-coding RNAs as regulators in epigenetics. Oncol. Rep..

[65] Neganova M.E., Klochkov S.G., Aleksandrova Y.R., Aliev G. (2022). Histone modifications in epigenetic regulation of cancer: Perspectives and achieved progress. Semin. Cancer Biol..

[66] Vafadar A., Shabaninejad Z., Movahedpour A., Mohammadi S., Fathullahzadeh S., Mirzaei H.R., Namdar A., Savardashtaki A., Mirzaei H. (2019). Long non-coding RNAs as epigenetic regulators in cancer. Curr. Pharm. Des..

[67] Arif K., Elliott E.K., Haupt L.M., Griffiths L.R. (2020). Regulatory mechanisms of epigenetic miRNA relationships in human cancer and potential as therapeutic targets. Cancers.

[68] Gulyaeva L.F., Kushlinskiy N.E. (2016). Regulatory mechanisms of microRNA expression. J. Transl. Med..

[69] Ha M., Kim V.N. (2014). Regulation of microRNA biogenesis. Nat. Rev. Mol. Cell Biol..

[70] Yi D., Xu L., Wang R., Lu X., Sang J. (2019). miR-381 overcomes cisplatin resistance in breast cancer by targeting MDR1. Cell Biol. Int..

[71] Cheng F., Zhao Z., Liu W. (2019). Long non-coding RNA ROR regulated ABCB1 to induce cisplatin resistance in osteosarcoma by sponging miR-153-3p. Eur. Rev. Med. Pharm. Sci..

[72] Li M., Gao M., Xie X., Zhang Y., Ning J., Liu P., Gu K. (2019). MicroRNA-200c reverses drug resistance of human gastric cancer cells by targeting regulation of the NER-ERCC3/4 pathway. Oncol. Lett..

[73] Shindo T., Niinuma T., Nishiyama N., Shinkai N., Kitajima H., Kai M., Maruyama R., Tokino T., Masumori N., Suzuki H. (2018). Epigenetic silencing of miR-200b is associated with cisplatin resistance in bladder cancer. Oncotarget.

[74] Yang H., Kong W., He L., Zhao J.-J., O’Donnell J.D., Wang J., Wenham R.M., Coppola D., Kruk P.A., Nicosia S.V. (2008). MicroRNA expression profiling in human ovarian cancer: miR-214 induces cell survival and cisplatin resistance by targeting PTEN. Cancer Res..

[75] Li L., Gu M., You B., Shi S., Shan Y., Bao L., You Y. (2016). Long non-coding RNA ROR promotes proliferation, migration and chemoresistance of nasopharyngeal carcinoma. Cancer Sci..

[76] Shi H., Pu J., Zhou X.-L., Ning Y.-Y., Bai C. (2017). Silencing long non-coding RNA ROR improves sensitivity of non-small-cell lung cancer to cisplatin resistance by inhibiting PI3K/Akt/mTOR signaling pathway. Tumor Biol..

[77] Sun Y.-F., Wang Y., Li X.-D., Wang H. (2022). SNHG15, a p53-regulated lncRNA, suppresses cisplatin-induced apoptosis and ROS accumulation through the miR-335-3p/ZNF32 axis. Am. J. Cancer Res..

[78] Liu R., Guo H., Lu S. (2018). MiR-335-5p restores cisplatin sensitivity in ovarian cancer cells through targeting BCL2L2. Cancer Med..

[79] Zampieri M., Ciccarone F., Calabrese R., Franceschi C., Bürkle A., Caiafa P. (2015). Reconfiguration of DNA methylation in aging. Mech. Ageing Dev..

[80] Flanagan J.M., Wilson A., Koo C., Masrour N., Gallon J., Loomis E., Flower K., Wilhelm-Benartzi C., Hergovich A., Cunnea P. (2017). Platinum-based chemotherapy induces methylation changes in blood DNA associated with overall survival in patients with ovarian cancer. Clin. Cancer Res..

[81] De Caceres I.I., Cortes-Sempere M., Moratilla C., Machado-Pinilla R., Rodriguez-Fanjul V., Manguan-Garcia C., Cejas P., López-Ríos F., Paz-Ares L., De Castrocarpeño J. (2010). IGFBP-3 hypermethylation-derived deficiency mediates cisplatin resistance in non-small-cell lung cancer. Oncogene.

[82] Toh T.B., Lim J.J., Chow E.K.-H. (2017). Epigenetics in cancer stem cells. Mol. Cancer.

[83] Easwaran H., Tsai H.-C., Baylin S.B. (2014). Cancer epigenetics: Tumor heterogeneity, plasticity of stem-like states, and drug resistance. Mol. Cell.

[84] Suresh R., Ali S., Ahmad A., Philip P.A., Sarkar F.H. (2016). The role of cancer stem cells in recurrent and drug-resistant lung cancer. Lung Cancer Pers. Med. Nov. Ther. Clin. Manag..

[85] Lopez-Ayllon B.D., Moncho-Amor V., Abarrategi A., de Cáceres I.I., Castro-Carpeño J., Belda-Iniesta C., Perona R., Sastre L. (2014). Cancer stem cells and cisplatin-resistant cells isolated from non-small-lung cancer cell lines constitute related cell populations. Cancer Med..

[86] Bora-Singhal N., Perumal D., Nguyen J., Chellappan S. (2015). Gli1-mediated regulation of Sox2 facilitates self-renewal of stem-like cells and confers resistance to EGFR inhibitors in non–small cell lung cancer. Neoplasia.

[87] Milewski D., Pradhan A., Wang X., Cai Y., Le T., Turpin B., Kalinichenko V.V., Kalin T.V. (2017). FoxF1 and FoxF2 transcription factors synergistically promote rhabdomyosarcoma carcinogenesis by repressing transcription of p21 Cip1 CDK inhibitor. Oncogene.

[88] Ran L., Chen Y., Sher J., Wong E.W., Murphy D., Zhang J.Q., Li D., Deniz K., Sirota I., Cao Z. (2018). FOXF1 defines the core-regulatory circuitry in gastrointestinal stromal tumor. Cancer Discov..

[89] Zhao J., Xue X., Fu W., Dai L., Jiang Z., Zhong S., Deng B., Yin J. (2020). Epigenetic activation of FOXF1 confers cancer stem cell properties to cisplatin-resistant non-small cell lung cancer. Int. J. Oncol..

[90] Yeon A., You S., Kim M., Gupta A., Park M.H., Weisenberger D.J., Liang G., Kim J. (2018). Rewiring of cisplatin-resistant bladder cancer cells through epigenetic regulation of genes involved in amino acid metabolism. Theranostics.

[91] Ha Y.-N., Sung H.Y., Yang S.-D., Chae Y.J., Ju W., Ahn J.-H. (2018). Epigenetic modification of α-N-acetylgalactosaminidase enhances cisplatin resistance in ovarian cancer. Korean J. Physiol. Pharmacol..

[92] Wang L., Xiang S., Williams K.A., Dong H., Bai W., Nicosia S.V., Khochbin S., Bepler G., Zhang X. (2012). Depletion of HDAC6 enhances cisplatin-induced DNA damage and apoptosis in non-small cell lung cancer cells. PLoS ONE.

[93] Cortes-Sempere M., De Miguel M., Pernia O., Rodriguez C., de Castro Carpeno J., Nistal M., Conde E., López-Ríos F., Belda-Iniesta C., Perona R. (2013). IGFBP-3 methylation-derived deficiency mediates the resistance to cisplatin through the activation of the IGFIR/Akt pathway in non-small cell lung cancer. Oncogene.

[94] Vera Puente O., Jiménez Hernández J., Pernía O., Rodriguez-Antolín C., Rodriguez C., Sanchez Cabo F., Soto Romero J., Rosas R., Lopez-Magallon S., Esteban Rodriguez I. (2017). DNA methylation of miR-7 is a mechanism involved in platinum response through MAFG overexpression in cancer cells. Theranostics.

[95] Chen L., Zhu Q., Lu L., Liu Y. (2020). MiR-132 inhibits migration and invasion and increases chemosensitivity of cisplatin-resistant oral squamous cell carcinoma cells via targeting TGF-β1. Bioengineered.

[96] Shi L., Xi J., Xu X., Peng B., Zhang B. (2019). MiR-148a suppressed cell invasion and migration via targeting WNT10b and modulating β-catenin signaling in cisplatin-resistant colorectal cancer cells. Biomed. Pharmacother..

[97] Vera O., Rodriguez-Antolin C., de Castro J., Karreth F.A., Sellers T.A., de Caceres I.I. (2018). An epigenomic approach to identifying differential overlapping and cis-acting lncRNAs in cisplatin-resistant cancer cells. Epigenetics.

[98] Li Z., Zhao L., Wang Q. (2016). Overexpression of long non-coding RNA HOTTIP increases chemoresistance of osteosarcoma cell by activating the Wnt/β-catenin pathway. Am. J. Transl. Res..

[99] Li F., Cao L., Hang D., Wang F., Wang Q. (2015). Long non-coding RNA HOTTIP is up-regulated and associated with poor prognosis in patients with osteosarcoma. Int. J. Clin. Exp. Pathol..

[100] Fan Y., Shen B., Tan M., Mu X., Qin Y., Zhang F., Liu Y. (2014). Long non-coding RNA UCA 1 increases chemoresistance of bladder cancer cells by regulating Wnt signaling. FEBS J..

[101] Zhou B., Zhuang X., Wang Y., Lin Z., Zhang D., Fan S., Huang Z., Li J., Chen W. (2017). Long non-coding RNA UCA1 increases chemoresistance of tongue cancer cells by regulating autophagy signaling. Int. J. Oral Maxillofac. Surg..

[102] Xu C., Guo Y., Liu H., Chen G., Yan Y., Liu T. (2018). TUG1 confers cisplatin resistance in esophageal squamous cell carcinoma by epigenetically suppressing PDCD4 expression via EZH2. Cell Biosci..

